# Predisposition to insulin resistance and obesity due to staple consumption of rice: Amylose content versus germination status

**DOI:** 10.1371/journal.pone.0181309

**Published:** 2017-07-20

**Authors:** Bilyaminu Abubakar, Norhasnida Zawawi, Abdul Rahman Omar, Maznah Ismail

**Affiliations:** 1 Laboratory of Molecular Biomedicine, Institute of Bioscience, Universiti Putra Malaysia, Serdang, Selangor, Malaysia; 2 Department of Pharmacology and Toxicology, Usmanu Danfodiyo University, Sokoto, Nigeria; 3 Department of Food Science, Faculty of Food Science and Technology, Universiti Putra Malaysia, Universiti Putra Malaysia, Serdang, Selangor, Malaysia; 4 Department of Animal Science, Faculty of Veterinary Medicine, Universiti Putra Malaysia, Serdang, Selangor, Malaysia; Hospital Infantil Universitario Nino Jesus, SPAIN

## Abstract

Type 2 diabetes is a metabolic disorder with established, well-defined precursors. Obesity and insulin resistance are amongst most important factors in predisposition to diabetes. Rice is a staple for about half the global population and its consumption has been strongly linked with diabetogenesis. We assert that tackling the prevalence of predisposing factors by modifying certain rice cultivars could reduce the global burden of obesity and insulin resistance, and by extension type 2 diabetes. Several rice cultivars with various properties were fed to nulliparous rats (five weeks old at the start of the experiment) for 90 days. They were then returned to a diet of standard pellets and mated with males raised on a standard diet. The resulting pups and dams were investigated for obesity and insulin resistance markers. We found that germination did more to reduce predisposition to obesity and insulin resistance than high amylose content. The combined reducing effect of germination and high amylose content on predisposition to obesity and insulin resistance was greater than the sum of their independent effects. Polished (white) rice with a low amylose content predisposed dams on a high-fat diet to markers of insulin resistance and obesity and this predisposition was inherited (in biochemical terms) by their F1 offspring. Overall, the results suggest that harnessing the beneficial properties of germination and amylose in rice would reduce the burden of obesity and insulin resistance, which are known to be key risk factors for development of type 2 diabetes.

## Introduction

The type 2 diabetes epidemic is fast reaching pandemic proportions: the number of people with the disease is approaching half a billion [1]. As with most metabolic diseases, the severity and onset of type 2 diabetes is strongly related to the pathogenicity of its precursors and/or risk factors [2]. Insulin resistance (IR) and obesity are amongst the most important risk factors for type 2 diabetes [3]. Epidemiological studies indicate that obesity resulting from IR may be the most important factor in the development and progression of type 2 diabetes [4]. The World Health Organisation (WHO) states that about 9 out of every 10 cases of type 2 diabetes are connected to excess weight and/or IR [1]. Since energy imbalance due to calorie overconsumption or disproportion is one of the front runners of obesity [5], modifying a staple food could do a lot to reduce the burden of type 2 diabetes via effects on obesity and IR.

Rice is a staple food for over 50% of the world population [6]. Remarkably, the countries where rice is a staple food–principally China and India - harbour more than half of the world’s type 2 diabetic population [7]. Several studies have suggested that consumption of white rice contributes strongly to development of type 2 diabetes [8],[9],[10]; however consumption of germinated brown rice has been shown to delay or prevent progression to type 2 diabetes from obesity and persistent glucose intolerance [11],[12],[13]. Earlier studies suggested that anti-obesity properties of germinated brown rice might be mediated partly by downregulation of lipogenic genes [14]. In addition, in 2004 Pawlak and co-researchers [15] demonstrated that a food’s glycaemic index - which in rice is partly a function of amylose content - influences how it affects glucose homeostasis and adiposity. Frei et al. [16] demonstrated that the amylose content of a rice cultivar affects its glycaemic index and hence the long-term, post-prandial course of blood glucose levels and lipid metabolism following consumption of rice. The aforementioned studies show that germination status and amylose content parameters could play an important role in progression to IR and obesity. A more recent study by Denardin et al [17] on rats demonstrated that the amylose content of a rice cultivar influences glycaemic and lipid metabolism.

In a bid to alleviate development of type 2 diabetes as a result of rice consumption, we explored the interactive and independent effects of amylose content and germination on the time course of development of obesity and IR, two key risk factors for development of type 2 diabetes. The interaction between these variables was tracked in rats whose mothers were exposed to dietary interventions.

## Methods

### Sample preparation

White and brown rice from MRQ 74 and MRQ 76 cultivars was provided by the Malaysian Agricultural Research and Development Institute (MARDI). The brown form of each cultivar was germinated as described previously [11] to produce the germinated brown cultivars. Briefly, the brown rice was washed and then soaked in 0.05% of sodium hypochlorite for 30 min before being washed with tap water; it was then soaked in 0.5% hydrogen peroxide for 6 h and dried overnight in an oven at 50°C. Antioxidant indices and phytochemicals including flavonoids, polyphenols, and some heavy metals were ascertained not to be of significant difference between the same forms (white or germinated) of the two rice cultivars.

### Proximate analysis and amylose content quantification

The proximate analysis of the rice types was performed as described previously [18], following the protocols of the Association of Official Analytical Chemists. Briefly, after determining the nitrogen content of samples using micro-Kjeldahl apparatus (KjeltechTM 2200 Auto Distillation Unit, FOSS Tecator, Hoganas, Sweden), their protein content was calculated as N × 5.95. The carbohydrate content was determined using the ashing process. Samples were incinerated in a furnace (Furnace 62700, Barnstead/Thermolyne, Dubuque, IA, USA) at 550°C and the their carbohydrate content calculated using the following formula: (100%–protein content–moisture content–ash content–crude fat content). All results were expressed as percentages of dry weight.

The amylose content of the rice cultivars was determined according to the International Standard Organisation protocol [19]. Briefly, 0.1 g of each sample, blank or standard, was weighed in triplicate and placed in a 100-ml volumetric flask to which 1 ml of 95% ethanol and 9 ml of 1M sodium hydroxide was added. The mixture was heated in a boiling water bath for 20 min and then allowed to cool to room temperature water was added to give a final volume of 100 ml. Next 0.5 ml of the test, standard or blank solution was pipetted into a 10-ml test tube containing 5 ml of water. Then 0.1 ml of 5% acetic acid and 0.2 ml of iodine (Lugol’s solution) were added to each test tube. Another 4.2 ml of water was added to each test tube to make the volume up to10 ml and the contents were thoroughly mixed using a vortex mixer. The resulting mixture was immediately measured at 720 nm against the blank using a spectrophotometer. A calibration curve was obtained and used to determine the percentage amylose content of the various rice cultivars.

### Animal handling

One hundred and twenty nulliparous, recently weaned Sprague-Dawley rats weighing 90–110 g were purchased from the animal house of the Faculty of Veterinary Medicine, University of Putra Malaysia, Serdang, Malaysia. The animals were housed in pairs in plastic cages, in a well-ventilated room with an approximate 12/12 h light/dark cycle at 25–30°C. Approval for the use of animals was sought from the Institutional Animal Care and Use Committee (IACUC) of the University of Putra Malaysia (Project approval number: UPM/IACUC/AUP- R017/2016). Animals were acclimatised to their new environment for 1 week, during which they were given standard rat chow *ad libitum* and water. The nutritional composition of the standard rat chow (Gold Coin, Port Klang, Malaysia) was as follows: 50% carbohydrate, 21% protein, 3% fat, 13% moisture, 8% ash and 5% fibre; the high-fat diet consisted of 50% standard rat chow, 24% ghee, 20% full-fat milk and 5% starch. Before the start of the experiment the fasting blood glucose concentration of the rats was determined on two separate occasions to confirm the absence of glucose intolerance.

### Interventions, anthropometry, dietary variables and biochemical analysis

The rats were divided into 20 groups (*n* = 6 per group) and assigned to various diets (Table 1). First the rats were fed the assigned experimental diet and water *ad libitum* for 90 days. Their weights and food intake were measured weekly. Rats with more than 10% body weights than the maximum body weights of rats fed on standard chow were considered as obese [20]. Oral glucose tolerance tests (OGTT; 2 g/kg dose of glucose) were carried out after days 7 and 77. Values for the homeostasis model of insulin resistance (HOMA-IR) were also determined after day 77 by collecting 0.5 ml of blood from the facial vein of 12-h fasted rats and using this to determine the fasting serum insulin and glucose levels using, respectively, an enzyme-linked immunosorbent assay (ELISA) and the glucose oxidase method. The fasting serum insulin and blood glucose levels were entered into an online HOMA-IR calculator to obtain the IR value.

**Table 1:** Dietary interventions used in the study.

Group	Diet	Protein (g/100 g)	Carbohydrate (g/100 g)	Fat (g/100 g)	Average Total calorie per (kcal/100 g)
**Standard chow-based interventions**					
SC	Standard chow (SC)	21.0	62.0	3.0	359
SC+50% HGBR	50% SC + 50% HAGBR	15.8	67.2	3.0	359
SC+50%LAGBR	50% SC + 50% LAGBR	15.9	66.7	4.4	370
SC+50%HAWR	50% SC + 50% HAWR	14.5	69.4	3.4	366
SC+50%LAWR	50% SC + 50% LAWR	14.4	68.9	3.3	363
SC+50%LAWR+AC	50% SC + 50% LAWR + AC	14.4	68.9	3.3	363
SC+25%HAGBR	75% SC + 25% HAGBR	18.5	64.6	2.9	358
SC+25%LAGBR	75% SC + 25% LAGBR	18.5	64.3	3.65	364
SC+25%HAWR	75% SC + 25% HAWR	17.8	65.7	3.15	362
SC+25%LAWR	75% SC + 25% LAWR	17.8	65.4	3.15	361
**High-fat diet-based interventions**					
HFD	High-fat diet (HFD)	22.5	46.6	30.6	552
HFD+50%HAGBR	HFD of which 50% of the pellet was substituted with HAGBR	18.0	49.2	30.6	544
HFD+50%LAGBR	HFD of which 50% of the pellet was substituted with LABGR	18.0	48.9	31.3	549
HFD+50%HAWR	HFD of which 50% of the pellet was substituted with HAWR	17.3	50.3	30.8	547
HFD+50%LAWR	HFD of which 50% of the pellet was substituted with LAWR	17.3	50.0	30.8	546
HFD+50%LAWR+AC	HFD of which 50% of the pellet was substituted with LAWR +AC	17.3	50.0	30.8	546
HFD+25%HAGBR	HFD of which 25% of the pellet was substituted with HAGBR	19.3	47.9	30.7	545
HFD+25%LAGBR	HFD of which 25% of the pellet was substituted with LAGBR	19.3	47.8	31.0	547
HFD+25%HAWR	HFD of which 25% of the pellet was substituted with HAWR	18.9	48.3	30.8	546
HFD+25%LAWR	HFD of which 25% of the pellet was substituted with LAWR	18.9	48.3	30.7	545

AC: acarbose (60 mg/kg); GBR: germinated brown rice; HAGBR: high amylose germinated brown rice; HAWR: high amylose white rice; HFD: high-fat diet; LAGBR: low amylose germinated brown rice; LAWR: low amylose white rice; SC: standard chow.

At the end of the 90-day treatment, the rats’ diet were reverted to standard rat chow and they were mated with male normoglycaemic rats. Following conception dams were housed individually. The weight and number of pups delivered by each dam was documented. Three weeks after weaning, pups and dams were fasted for 12 h and then 1 ml blood was collected from each rat by cardiac puncture under xylazine and ketamine anaesthesia (10 mg/kg:100 mg/kg). The blood was kept in plain tubes and allowed to clot while standing. The clotted blood was spun at 5000 rpm for 5 min and then the separated serum was split into aliquots and the levels of adiponectin, leptin and insulin were determined using ELISA kits according to the manufacturer’s protocol (Millipore, USA). Levels of low-density lipoprotein (LDL), high-density lipoprotein (HDL), total cholesterol (TC) and triacyl glycerol (TG) were determined using a Siemens automatic chemistry analyser (Dimension Xpand Plus Integrated Chemistry System).

### Statistical analysis

Results were represented as mean ± standard deviation (SD). Statistical analysis was carried out using Minitab 16. One-way analysis of variance (ANOVA) or general regression analysis was used, as appropriate. Tukey’s test was used for *post hoc* analysis of differences detected using one-way ANOVA. The significance level was set at *p* < 0.05. Values are represented in tables and figures.

## Results

Table 1 displays the energy distribution of the various diet interventions. The energy content (kcal/100 g) for the standard chow (SC) based diet groups ranged from 359 kcal/100 g to 370 kcal/100 g. The difference among the energy contents of the high-fat diet interventions was 8 kcal/100 g. The proximate analyses of the two rice cultivars are presented in Table 2. The carbohydrate content ranged from 71.28% to 76.72%. The amylose content of MRQ 74 rice - both the white form and the germinated brown form - was more than double that of MRQ 76, making MRQ 74 a high-amylose rice and MRQ 76 a low-amylose rice. The two rice types had different proximate compositions. In the two tests used to ascertain the rats’ glucose tolerance, fasting blood glucose levels were in the range 3.9-5.2 mmol/L, as shown in S1 and S2 Tables. These values were within the accepted normal range.

**Table 2:** Proximate composition and percentage amylose composition of the rice cultivars (g/100 g DM basis).

Rice cultivar	Composition (mean ± SD)									
	Ash	Moisture		Fat	Protein		Carbohydrates	Calorie(Kcal)	Amylose (%)	
MRQ 74 wr	0.56 ± 0.009^c^	12.89 ± 0.5^a^		3.75 ± 0.2^b^	8.08 ± 0.7^b^		76.72 ± 0.1^a^	356.93 ± 0.8^d^	26.1 ± 1.4^a^	
MRQ 74 gbr	1.55 ± 0.015^a^	11.45 ± 0.5^bc^		2.90 ± 0.1^c^	10.69 ± 0.5^a^		72.43 ± 0.5^c^	362.10 ± 0.6^c^	25.7 ± 2.1^a^	
MRQ 76 wr	0.34 ± 0.009^d^	12.39 ± 0.3^ab^		3.60 ± 0.2^b^	7.83 ± 0.3^b^		75.85 ± 0.3^b^	367.12 ± 0.7^b^	12.5 ± 1.3^b^	
MRQ 76 gbr	1.47 ± 0.010^b^	10.57 ± 0.6^c^		5.85 ± 0.1^a^	10.83 ± 0.7^a^		71.28 ± 0.2^d^	381.09 ± 0.8^a^	12.5 ± 0.9^b^	

Mean values within a column superscripted by the same letter are not significantly different at p < 0.05. wr: white rice; gbr: germinated brown rice; SD: standard deviation.

### Anthropometry and dietary intake: Standard chow-based interventions

**Fig 1:**
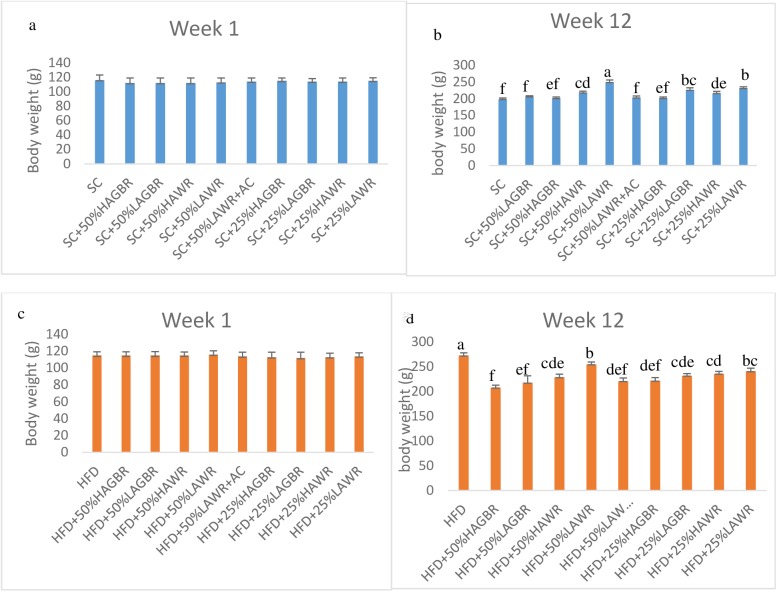
Body weights of rats fed on standard chow-based diet (a and b) and high-fat based diet (c and d) at weeks 1 and 12. Bars with different letters denotes values that are significantly different (p<0.05) in Tukey’s multiple comparison test. AC: acarbose; GBR: germinated brown rice; HAGBR: high amylose germinated brown rice; HAWR: high amylose white rice; HFD: high-fat diet LAGBR: low amylose germinated brown rice; LAWR: low amylose white rice; SC: standard chow.

Changes in the rats’ body weights are presented below in Fig 1. All groups had similar body weights at the start of the experiment, but differences emerged as the experiment progressed. After the twelfth week of treatment both low-amylose white rice (LAWR) groups (50% and 25%) were more than 10% heavier than the standard-chow control group (obese). In contrast, the rats fed a diet consisting of 50% germinated low-amylose rice had similar body weights to the standard chow group. Rats fed the lower dose (25%) of germinated low-amylose brown rice did, however, gain more weight than those on the standard chow diet. Acarbose prevented the extra weight gain associated with a high dose of LAWR: rats fed on LAWR plus acarbose had similar body weights to those fed standard chow. All rats fed with high-amylose rice - whether white or germinated - had lower body weights than counterparts fed with low-amylose rice. All the pups were of similar weights until the end of the study as shown in S3 Table.

### Anthropometry and dietary intake: High-fat diet-based interventions

Anthropometric data from rats fed the various high-fat diets are presented in Fig 1. At the start of the interventions all the groups had similar body weights. After the twelfth week of treatment the 100% high-fat diet (HFD) group was heavier than all the other groups. All the groups eating germinated rice, except those on the 25% low-amylose rice diet, had lower body weights than the group fed white (polished) rice. The only high-amylose rice group to have lower body weight than the low-amylose rice groups was the group fed a diet consisting of 50% germinated rice. Acarbose reduced the increase in body weight associated with the 50% LAWR diet. Overall, including germinated or high-amylose rice in the diet tended to reduce rats’ body weight, whereas white rice and low-amylose rice had the opposite effect. All the pups were of similar weight until the end of the study, as shown in S4 Table.

### Oral glucose tolerance test (OGTT) and homeostasis model assessment for insulin resistance (HOMA-IR)

**Fig 2:**
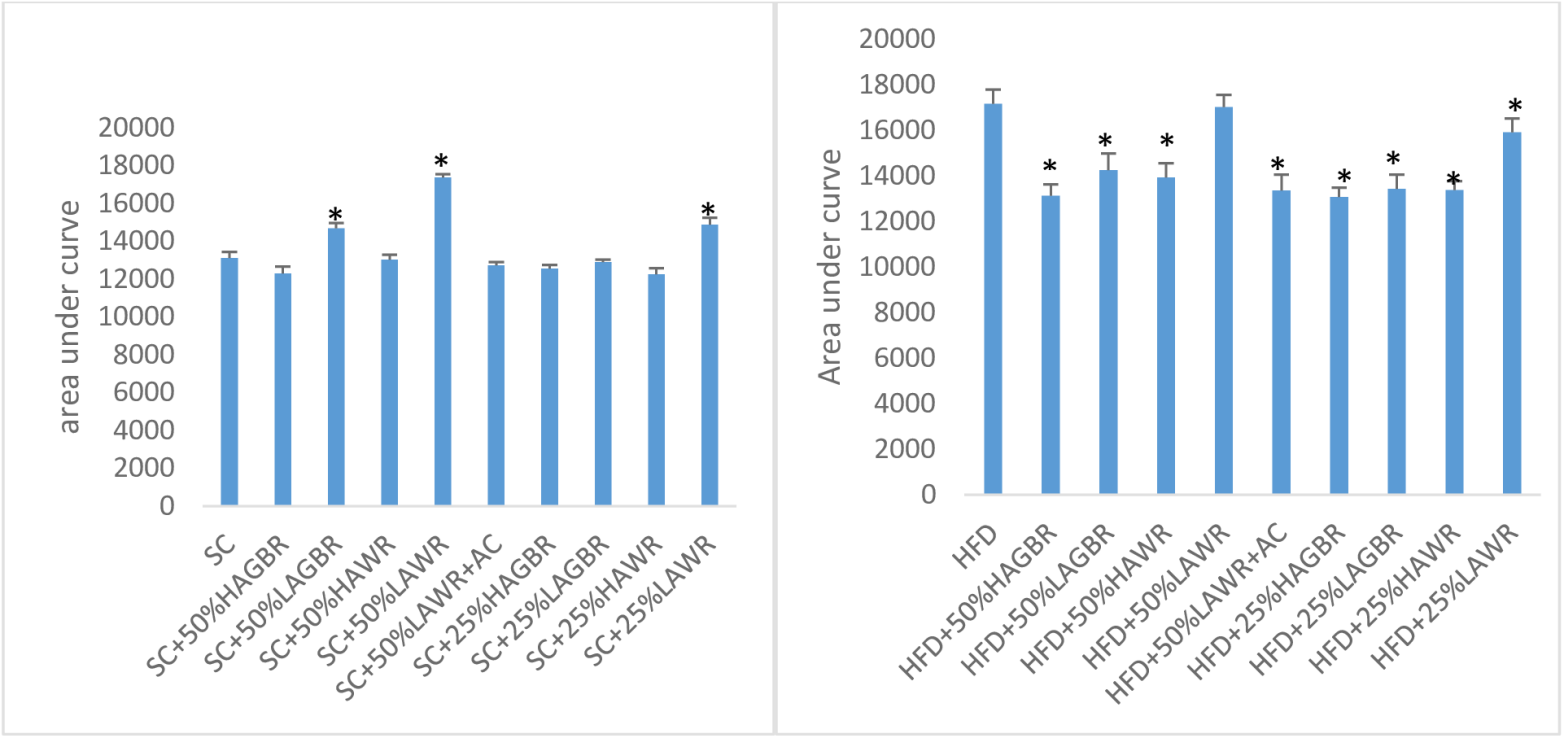
2 h area under curve after oral glucose tolerance test on rats fed with different rice interventions. Bars with asterisk denotes values that are significantly different (p<0.05) in Dunett’s multiple comparison test from the control (SC and HFD groups). AC: acarbose; GBR: germinated brown rice; HAGBR: high amylose germinated brown rice; HAWR: high amylose white rice; HFD: high fat diet; LAGBR: low amylose germinated brown rice; LAWR: low amylose white rice; SC: standard chow.

In the first OGTT (after day 7) all rats had normal glucose tolerance. The area under curve (AUC) results from the second OGTT (after day 77), which was carried out before the dams conceived, are presented below in Fig 2.

#### Standard chow-based interventions

All the dams fed low-amylose rice had higher AUC values than the standard-chow control group, with the exception of LAWR-fed rats who received a daily dose of acarbose. Dams fed a diet consisting of 50% LAWR had the highest AUC values. All dams fed with white rice - irrespective of amylose content - had higher IR values than dams fed standard chow (Table 3). The acarbose positive control group had similar IR to the standard-chow control group. The 50% LAWR group had the highest IR and, as expected, the lowest insulin sensitivity. All the pups had similar AUC values until the end of the study (S1 Fig).

**Table 3:** Insulin resistance status of dams and their pups.

Group	Steady state beta cell function (%B)	Insulin sensitivity (%S)	Insulin resistance
**Standard chow-based interventions**			
SC Dams	103.4 ± 21.6^abc^	125.2 ± 4.8 ^bc^	0.80 ± 0.13^def^
SC pups	104.3 ± 18.4^a^	114.1 ± 8.1^bc^	0.88 ± 0.06^bcd^
SC+50%HAGBR dams	88.1 ± 8.2^abc^	113.3 ± 15.1^bcd^	0.89 ± 0.11^cde^
SC+50%HAGBR pups	99.4 ± 18.4^a^	120.2 ± 4.6^bc^	0.83 ± 0.03^cde^
SC+50%LAGBR dams	78.3 ± 1.6^bc^	176.0 ± 22.3^a^	0.58 ± 0.07^f^
SC+50%LAGBR pups	114.4 ± 6.8^a^	146.1 ± 13.0^ab^	0.69 ± 0.06^de^
SC+50%HAWR dams	135.7 ± 25.4^a^	94.1 ± 1.9^d^	1.06 ± 0.12^c^
SC+50%HAWR pups	101.4 ± 12.1^a^	117.5 ± 8.1^bc^	0.85 ± 0.05^bcde^
SC+50%LAWR dams	67.9 ± 44.2^c^	41.1 ± 1.9^e^	2.44 ± 0.12^a^
SC+50%LAWR pups	80.9 ± 3.9^a^	55.9 ± 1.4^d^	1.79 ± 0.05^a^
SC+50%LAWR+AC dams	86.8 ± 10.7^abc^	141.7 ± 14.5^b^	0.71 ± 0.07^ef^
SC+50%LAWR+AC pups	105.1 ± 10.5^a^	153.9 ± 13.3^a^	0.65 ± 0.05^e^
SC+25%HAGBR dams	92.9 ± 3.6^abc^	101.4 ± 3.1^cd^	0.99 ± 0.13^cd^
SC+25%HAGBR pups	82.2 ± 5.9^a^	121.7 ± 9.7^abc^	0.83 ± 0.07^cde^
SC+25%LAGBR dams	82.3 ± 7.5^abc^	114.5 ± 6.4^bcd^	0.87 ± 0.05^cde^
SC+25%LAGBR pups	97.5 ± 21.8^a^	136.5 ± 22.4^ab^	0.74 ± 0.11^de^
SC+25%HAWR dams	130.6 ± 16.2^ab^	95.6 ± 3.5^d^	1.05 ± 0.04^c^
SC+25%HAWR pups	128.3 ± 38.3^a^	101.3 ± 12.7^c^	1.00 ± 0.12^bc^
SC+25%LAWR dams	84.3 ± 4.3^abc^	50.0 ± 3.1^e^	2.00 ± 0.13^b^
SC+25%LAWR pups	125.4 ± 33.1^a^	95.9 ± 5.0^c^	1.04 ± 0.05^b^
**High-fat diet-based interventions**			
HFD Dams	106.9 ± 3.4^ab^	32.8 ± 0.3^d^	3.05 ± 0.04^ab^
HFD pups	125.3 ± 48.9	38.0 ± 10.6^d^	2.81 ± 0.32^a^
HFD+50%HAGBR dams	73.6 ± 7.3^d^	153.4 ± 13.2^a^	0.65 ± 0.05^d^
HFD+50%HAGBR pups	85.6 ± 7.8^ab^	153.9 ± 9.4^a^	0.65 ± 0.04^b^
HFD+50%LAGBR dams	69.1 ± 24.5^cd^	84.4 ± 2.3^b^	1.18 ± 0.03^cd^
HFD+50%LAGBR pups	79.1 ± 10.8^b^	110.3 ± 3.4^b^	0.91 ± 0.02^b^
HFD+50%HAWR dams	150.7 ± 34.3^cd^	72.1 ± 20.9^bc^	1.45 ± 0.36^cd^
HFD+50%HAWR pups	109.5 ± 22.3^ab^	115.0 ± 4.3^b^	0.87 ± 0.03^b^
HFD+50%LAWR dams	93.9 ± 21.2^a^	31.9 ± 12.6^d^	3.43 ± 1.10^a^
HFD+50%LAWR pups	88.7 ± 7.5^ab^	39.7 ± 1.7^d^	2.52 ± 0.11^a^
HFD+50%LAWR+AC dams	106.9 ± 14.3^c^	55.1 ± 1.6^cd^	1.81 ± 0.05^c^
HFD+50%LAWR+AC pups	81.2 ± 2.9^b^	129.3 ± 10.3^b^	0.78 ± 0.06^b^
HFD+25%HAGBR dams	100.2 ± 4.4^cd^	71.6 ± 2.3^bc^	1.39 ± 0.04^cd^
HFD+25%HAGBR pups	122.9 ± 29.6^ab^	111.5 ± 8.0^b^	0.90 ± 0.06^b^
HFD+25%LAGBR dams	123.4 ± 20.1^cd^	60.9 ± 5.2^bc^	1.65 ± 0.13^cd^
HFD+25%LAGBR pups	128.3 ± 48.8^ab^	67.7 ± 5.0^c^	1.48 ± 0.11^b^
HFD+25%HAWR dams	176.6 ±39.3^bc^	48.3 ± 2.7^cd^	2.07 ± 0.12^bc^
HFD+25%HAWR pups	99.1 ± 21.1^ab^	83.4 ± 6.5^c^	1.20 ± 0.09^b^
HFD+25%LAWR dams	89.6 ± 4.2^bc^	49.3 ± 1.4^cd^	2.03 ± 0.05^bc^
HFD+25%LAWR pups	157.4 ± 6.3^a^	84.8 ± 14.4^c^	1.20 ± 0.18^b^

Data represent mean ± SD (n = 5). Different alphabet in each column for dams and pups separately denotes significant difference (p < 0.05) in Tukey’s multiple comparison test. AC: acarbose; GBR: germinated brown rice; HAGBR: high amylose germinated brown rice; HAWR: high amylose white rice; HFD: high-fat diet; LAGBR: low amylose germinated brown rice; LAWR: low amylose white rice; SC: standard chow

#### High-fat diet-based interventions

With the exception of the LAWR-fed dams, all dams had lower AUC values than those fed a 100% HFD. Increasing the dose of LAWR from 25% to 50% increased the AUC value. All dams, except the LAWR-fed rats, had lower IR than the group fed a 100% HFD. IR in the pups followed a similar pattern to IR in the mothers. Irrespective of the germination status, all dams fed high-amylose rice - and their pups ‒ had lower IR than their low-amylose counterparts, with the exception of the group fed a diet consisting of 25% high-amylose white rice. Only pups from mothers fed a 100% HFD or a 50% LAWR diet had IR outside the normal range. All the pups had similar AUC values until the end of the study (S2 Fig)

### Analysis of lipid profiles and adipocytokine levels

The lipid profile and adipocytokine data are presented in Table 4 below.

**Table 4:** Lipid profile and adipocytokine levels of dams and pups in the various standard-chow and high-fat diet groups.

Group	adp lep		tc tg ldl hdl			
	ng/ml		mmol/L			
**Standard chow-based interventions**						
SC Dams	93.28 ± 9.3^a^	0.355 ± 0.09^a^	1.30 ± 0.16^ab^	0.37 ± 0.03^c^	0.28 ± 0.03^f^	0.28 ± 0.03^a^
SC pups	78.68 ± 6.5^ab^	0.370 ± 0.08^a^	1.10 ± 0.16^ab^	0.34 ± 0.04^a^	0.28 ± 0.03^a^	0.27 ± 0.02^a^
SC+50%HAGBR dams	84.56 ± 6.3^ab^	0.348 ± 0.09^a^	1.27 ± 0.05^ab^	0.37 ± 0.02^c^	0.28 ± 0.02^f^	0.28 ± 0.02^a^
SC+50%HAGBR pups	81.23 ± 6.4^ab^	0.368 ± 0.09^a^	1.37 ± 0.09^ab^	0.33 ± 0.02^a^	0.29 ± 0.03^a^	0.28 ± 0.03^a^
SC+50%LAGBR dams	73.67 ± 10.3^c^	0.376 ± 0.08^a^	1.07 ± 0.31^ab^	0.30 ± 0.02^c^	0.30 ± 0.01^f^	0.29 ± 0.03^a^
SC+50%LAGBR pups	79.84 ± 6.7^ab^	0.336 ± 0.07^a^	1.40 ± 0.29^a^	0.30 ± 0.01^a^	0.30 ± 0.02^a^	0.31 ± 0.01^a^
SC+50%HAWR dams	56.87 ± 5.1^d^	0.364 ± 0.09^a^	1.43 ± 0.12^ab^	0.40 ± 0.05^c^	0.49 ± 0.01^bc^	0.30 ± 0.01^a^
SC+50%HAWR pups	67.57 ± 5.7^bcd^	0.379 ± 0.09^a^	1.40 ± 0.14^a^	0.30 ± 0.02^a^	0.28 ± 0.02^a^	0.29 ± 0.02^a^
SC+50%LAWR dams	38.16 ± 3.9^e^	0.515 ± 0.14^a^	1.67 ± 0.09^a^	0.74 ± 0.06^a^	0.60 ± 0.01^a^	0.28 ± 0.02^a^
SC+50%LAWR pups	41.06 ± 2.5^e^	0.363 ± 0.08^a^	1.30 ± 0.08^ab^	0.34 ± 0.05^a^	0.27 ± 0.02^a^	0.27 ± 0.02^a^
SC+50%LAWR+AC dams	74.86 ± 6.3^c^	0.358 ± 0.10^a^	1.27 ± 0.25^ab^	0.57 ± 0.01^b^	0.49 ± 0.01^bc^	0.28 ± 0.01^a^
SC+50%LAWR+AC pups	87.91 ± 7.1^a^	0.373 ± 0.09^a^	1.13 ± 0.12^ab^	0.36 ± 0.03^a^	0.28 ± 0.01^a^	0.29 ± 0.03^a^
SC+25%HAGBR dams	76.18 ± 6.3^bc^	0.375 ± 0.09^a^	1.03 ± 0.17^b^	0.39 ± 0.02^c^	0.39 ± 0.01^e^	0.29 ± 0.03^a^
SC+25%HAGBR pups	77.31 ± 6.2^bc^	0.381 ± 0.08^a^	0.87 ± 0.09^b^	0.31 ± 0.01^a^	0.30 ± 0.01^a^	0.30 ± 0.01^a^
SC+25%LAGBR dams	53.94 ± 5.5^d^	0.494 ± 0.10^a^	1.37 ± 0.17^ab^	0.37 ± 0.03^c^	0.41 ± 0.01^de^	0.30 ± 0.03^a^
SC+25%LAGBR pups	55.19 ± 5.2^de^	0.375 ± 0.08^a^	1.30 ± 0.14^ab^	0.30 ± 0.02^a^	0.30 ± 0.01^a^	0.29 ± 0.01^a^
SC+25%HAWR dams	56.07 ± 4.7^d^	0.372 ± 0.08^a^	1.27 ± 0.17^ab^	0.40 ± 0.02^c^	0.46 ± 0.01^cd^	0.30 ± 0.01^a^
SC+25%HAWR pups	63.76 ± 5.2^cd^	0.402 ± 0.10^a^	1.33 ± 0.12^ab^	0.38 ± 0.01^a^	0.28 ± 0.02^a^	0.25 ± 0.04^a^
SC+25%LAWR dams	49.20 ± 3.9^d^	0.441 ± 0.11^a^	1.37 ± 0.05^ab^	0.33 ± 0.02^c^	0.52 ± 0.01^b^	0.29 ± 0.01^a^
SC+25%LAWR pups	62.00 ± 5.1^cd^	0.371 ± 0.08^a^	1.40 ± 0.08^a^	0.31 ± 0.01^a^	0.29 ± 0.03^a^	0.28 ± 0.02^a^
**High-fat diet-based interventions**						
HFD Dams	26.60 ± 2.0^f^	1.980 ± 0.79^a^	2.20 ± 0.00^a^	1.10 ± 0.03^b^	0.91 ± 0.01^a^	0.28 ± 0.02^a^
HFD pups	31.86 ± 3.2^e^	0.412 ± 0.11^a^	1.50 ± 0.08^ab^	0.60 ± 0.02^a^	0.45 ± 0.02^a^	0.28 ± 0.02^a^
HFD+50%HAGBR dams	83.93 ± 4.5^a^	0.335 ± 0.07^b^	1.07 ± 0.05^e^	0.42 ± 0.02^e^	0.33 ± 0.02^g^	0.28 ± 0.02^a^
HFD+50%HAGBR pups	85.75 ± 4.2^ab^	0.385 ± 0.10^a^	0.97 ± 0.09^bc^	0.30 ± 0.01^c^	0.27 ± 0.01^b^	0.28 ± 0.02^a^
HFD+50%LAGBR dams	57.78 ± 4.1^cd^	0.406 ± 0.08^b^	1.63 ± 0.09^cd^	0.62 ± 0.03^d^	0.45 ± 0.02^f^	0.28 ± 0.02^a^
HFD+50%LAGBR pups	69.93 ± 4.9^abcd^	0.366 ± 0.09^a^	1.37 ± 0.19^abc^	0.34 ± 0.04^c^	0.29 ± 0.03^b^	0.28 ± 0.01^a^
HFD+50%HAWR dams	47.21 ± 2.8^de^	0.368 ± 0.08^b^	1.67 ± 0.05^bc^	0.90 ± 0.01^c^	0.72 ± 0.01^c^	0.26 ± 0.02^a^
HFD+50%HAWR pups	68.73 ± 3.6^bcd^	0.401 ± 0.10^a^	1.20 ± 0.29^abc^	0.46 ± 0.05^b^	0.33 ± 0.03^b^	0.26 ± 0.02 ^a^
HFD+50%LAWR dams	21.49 ± 1.9^f^	2.992 ± 1.15^a^	2.13 ± 0.34^ab^	1.30 ± 0.06^a^	0.82 ± 0.02^b^	0.30 ± 0.00^a^
HFD+50%LAWR pups	29.32 ± 2.1^e^	0.473 ± 0.12^a^	1.60 ± 0.16^a^	0.68 ± 0.01^a^	0.47 ± 0.02^a^	0.30 ± 0.00^a^
HFD+50%LAWR+AC dams	66.57 ± 3.2^bc^	0.377 ± 0.07^b^	1.20 ± 0.08^cde^	0.93 ± 0.04^c^	0.71 ± 0.02^c^	0.26 ± 0.02^a^
HFD+50%LAWR+AC pups	88.28 ± 5.3^a^	0.389 ± 0.10^a^	1.07 ± 0.05^abc^	0.34 ± 0.02^c^	0.30 ± 0.01^b^	0.27 ± 0.02^a^
HFD+25%HAGBR dams	75.41 ± 4.7^ab^	0.349 ± 0.07^b^	1.27 ± 0.09^cde^	0.68 ± 0.01^d^	0.53 ± 0.01^e^	0.26 ± 0.04^a^
HFD+25%HAGBR pups	84.08 ± 5.3^abc^	0.360 ± 0.07^a^	0.90 ± 0.08^bc^	0.37 ± 0.02^bc^	0.27 ± 0.02^b^	0.24 ± 0.01^a^
HFD+25%LAGBR dams	55.48 ± 3.1^de^	0.382 ± 0.10^b^	1.17 ± 0.17^de^	0.84 ± 0.04^c^	0.55 ± 0.02^e^	0.30 ± 0.01^a^
HFD+25%LAGBR pups	76.11 ± 5.5^abc^	0.378 ± 0.09^a^	1.27 ± 0.34^abc^	0.31 ± 0.02^c^	0.30 ± 0.01^b^	0.30 ± 0.01^a^
HFD+25%HAWR dams	46.88 ± 4.2^e^	0.385 ± 0.07^b^	1.47 ± 0.05^cde^	0.85 ± 0.01^c^	0.65 ± 0.02^d^	0.29 ± 0.01^a^
HFD+25%HAWR pups	66.65 ± 4.3^cd^	0.365 ± 0.08^a^	0.87 ± 0.05^c^	0.33 ± 0.03^c^	0.31 ± 0.01^b^	0.30 ± 0.01^a^
HFD+25%LAWR dams	45.26 ± 3.9^e^	0.450 ± 0.11^b^	1.57 ± 0.05^cd^	0.95 ± 0.04^c^	0.73 ± 0.01^c^	0.26 ± 0.03^a^
HFD+25%LAWR pups	54.40 ± 3.3^d^	0.377 ± 0.08^a^	1.17 ± 0.09^abc^	0.36 ± 0.04^bc^	0.29 ± 0.02^b^	0.26 ± 0.03^a^

Data represent mean ± SD (n = 5). Different alphabet in each column for dams and pups separately denotes significant difference (p < 0.05) in Tukey’s multiple comparison test. AC: acarbose; ADP: adiponectin; GBR: germinated brown rice; HAGBR: high amylose germinated brown rice; HAWR: high amylose white rice; HFD: high-fat diet; HDL: high-density lipoprotein; LAGBR: low amylose germinated brown rice; LAWR: low amylose white rice; LDL: low-density lipoprotein; LEP: leptin; SC: standard chow; TC: total cholesterol; TG: triacylglycerol.

#### Standard chow-based interventions

All pups and dams had similar leptin values. In the dams, all germinated rice cultivars improved serum adiponectin levels relative to the standard-chow control group, irrespective of amylose content. Similar to effect of germination status on adiponectin levels of the dams, amylose content also improved adiponectin levels. The dams fed on 50% high-amylose germinated brown rice (HAGBR) diet and their pups had comparable adiponectin levels to the dams and pups of the control groups. The dams and pups of all groups had similar HDL levels. There were group differences in LDL levels amongst the dams, with the 50% LAWR group having the highest LDL level; there were no group differences in LDL level amongst the pups. All the dams had similar TG levels, except those in the 50% LAWR group and the positive control (acarbose) group. All the pups had similar serum TG levels. Dams associated with 50% HAWR and 25% HAGBR had dissimilar serum TC values when compared to their counterparts in other groups.

#### High-fat diet-based interventions

All the groups of dams and pups had similar HDL levels. Dams fed on a 100% HFD, those fed on a 50% LAWR and their corresponding pups had higher LDL values compared to their corresponding counterparts in other groups. The dams of the 50% LAWR group had the lowest adiponectin levels. Amylose content and germination had independent effects on the dams’ TC and TG levels. Dams in the 100% HFD and the 50% LAWR groups had higher leptin levels than the dams in the other groups. All pups had similar leptin levels. There were group differences in adiponectin levels amongst both dams and pups. The dams fed on a 50% LAWR and those fed on a 100% HFD had lower adiponectin levels than their counterparts. The adiponectin levels of the pups from these groups (50% LAWR and 100% HFD), were also higher than those of their counterparts. The dams with the highest adiponectin levels were those in the acarbose-treated positive control group and those fed diets consisting of 50% or 25% HAGBR.

Table 5 displays regression coefficients and interaction effect of amylose content and germination status for the various rice cultivars. In general, the individual effects of germination status and amylose content on the IR and obesity markers were stronger than the interaction effects.

**Table 5:** Regression and interaction coefficients of amylose content and germination status of rice on various markers of insulin resistance and obesity in rats.

Obesity and/or IR marker	Regression coefficient	Amylose status/ germination status interaction	Obesity and/ or IR marker	Regression coefficient	Amylose content/ germination status interaction
**SC based interventions**			**HFD based interventions**		
Weight X SC dams	0.000*	0.000*	Weight X HFD dams	0.000*	0.000*
AUC X SC dams after wk 11	0.000*	0.000*	AUC X HFD dams after wk 11	0.000*	0.000*
ADP X SC dams	0.000*	0.048*	ADP X HFD dams	0.000*	0.937
ADP X SC pups	0.000*	0.001*	ADP X HFD pups	0.000*	0.038*
LEP X SC dams	0.250	0.330	LEP X HFD dams	0.001*	0.005*
LEP X SC pups	0.928	0.865	LEP X HFD pups	0.624	0.470
TC X SC dams	0.045*	0.117	TC X HFD dams	0.003*	0.703
TC X SC pups	0.929	0.604	TC X HFD pups	0.064	1.000
TG X SC dams	0.000	0.000	TG X HFD dams	0.000*	0.002*
TG X SC pups	0.539	0.169	TG X HFD pups	0.001*	0.005*
LDL X SC dams	0.000	0.006	LDL X HFD dams	0.000*	0.685
LDL X SC pups	0.572	0.388	LDL X HFD pups	0.000*	0.004*
HDL X SC dams	0.879	0.479	HDL X HFD dams	0.225	0.139
HDL X SC pups	0.334	0.130	HDL X HFD pups	0.225	0.139
IR X SC dams	0.000*	0.000*	IR X HFD dams	0.001*	0.064
IR X SC pups	0.000*	0.000*	IR X HFD pups	0.000*	0.000*

Data represent *p*-value results after general regression analysis. Significant regression coefficient or interaction effect are denoted with the asterisk sign (*p* ≤ 0.05). ADP: adiponectin; AUC: area under curve; HFD: high fat diet; HDL: high-density lipoprotein; IR: insulin resistance; LDL: low-density lipoprotein; LEP: leptin; SC: standard chow; OGTT: oral glucose tolerance test; TC: total cholesterol; TG: triacylglycerol.

## Discussion

Two sets of interventions were used in this study. The standard chow-based interventions were used to assess how the choice of rice cultivar could affect the propensity to obesity and IR of individuals who consume rice on a daily basis. The HFD-based interventions were used to assess how choice of rice cultivar could affect the propensity to obesity and IR of individuals who consume rice on a daily basis in the context of a diet designed to result in weight gain (a high-fat, high-calorie diet). Other predisposing factors such as genetics were largely controlled - all rats shared the same genealogy. The results of the standard chow-based interventions demonstrate how the amylose content and germination status of the rice cultivar consumed influence onset of risk factors for metabolic diseases. The recent paradigm shift in research on diabetes, from focusing on dietary fat to recognising that dietary carbohydrate is a very important contributor to obesity and IR [21], gives credence to the idea that the starch content of rice (amylose/amylopectin) is important, especially for those who consume rice as a staple. The HFD-based interventions demonstrate how dietary fat accelerates the onset of risk factors for obesity and IR and how the choice of rice cultivar influences their onset.

In the context of a diet based on standard chow both doses of LAWR influenced the body weight of dams. Fig 1A and 1B illustrate how the amylose content of rice was related to weight gain. Percentage amylose content influenced the weights of the rats when other parameters were kept constant (Fig 1B). Difference in percentage dietary amylose/amylopectin composition has been linked to alterations in glucose metabolism in normal and diabetic rats [22]. Dams fed low-amylose rice, whether in the context of a diet based on standard chow or a HFD, had higher weights than their high-amylose rice-fed counterparts. It has been proposed that the body weight differences associated with consumption of rice cultivars with differing amylose content of the rats is due to formation of a lipid-amylose complex that retards maximal intestinal absorption of amylose. This is not the case for amylopectin [23]. It is also due to the absence in amylose, of an easily cleavable alpha 1–6 glycosidic bond (branching). Amylopectin, on the other hand, is easily digested and rapidly absorbed due to the presence of alpha 1–6 glycosidic bond in its structure, thereby enhancing maximal postprandial glycaemia [24].

Irrespective of germination status, the low-amylose rice cultivars were associated with a high plasma glucose response (AUC values) in both the HFD- and standard chow-based interventions. AUC values were correlated with IR (*r*^2^ = 69.6%, *p* = 0.017). The results suggest that there is a negative association between a rice cultivar’s amylose content and development of IR in dams fed that rice cultivar over a long period. Chronic plasma glucose overload (large AUC values) ‒ as displayed by the dams in the low-amylose rice groups ‒ can provoke sustained hyperinsulinaemia and/or hyperglycaemia which in turn, may result in diet-induced obesity and IR [25]. Acarbose is an anti-hyperglycaemic drug that acts by competitively inhibiting intestinal α-glucosidase and pancreatic α-amylase, thus reducing the magnitude of postprandial changes in plasma glucose level. Dams given acarbose alongside a diet including LAWR had lower AUC values than dams fed LAWR without acarbose, which provides further support for our claim that consumption of LAWR might be linked to IR via chronic hyperglycaemia and weight gain. Doubling the dose of LAWR increased the AUC values in dams in the context of both the HFD and the standard chow-based diet. Germination of a rice cultivar appears to prevent excessive weight gain as germination reduced the effects of consumption of low-amylose rice; this is apparent from a comparison of the effect on weight of the low-amylose germinated brown rice (LAGBR) and LAWR groups. The obeso-protective effects of high amylose content and germination appear to be additive, as consumption of a germinated, high-amylose rice cultivar was associated with the most favourable AUC and IR values.

As the amylose content and germination status of the rice cultivars influenced rats’ weight as detailed above, the associations between adiponectin levels (in both sets of interventions and both generations) and amylose content and germination status may be due to the influence of these factors on adiposity as this adipocytokine is secreted exclusively by adipocytes. Leptin level was not associated with the germination status or amylose content of the rice cultivars when fed in the context of a diet based on standard chow. However, in the context of a HFD leptin levels in dams were associated with the germination status or amylose content of the rice cultivars. Several mechanisms have been proposed for the reduction of TG, LDL and TC levels and body weights associated with consumption of germinated rice including downregulation of lipogenic genes [26],[27]. Germination reduces the build-up of fatty acids (observed lipid profile reduction) from *de novo* lipogenesis - which impairs insulin signalling partly through inflammatory processes [28] - and this is partly responsible for its retardant effect on IR. Pups inherited markers of dyslipidaemia (high TG, LDL and TC levels), obesity (apparent leptin and adiponectin levels) and IR, although these effects were not associated with increased body weight or changes in AUC values and this provides evidence that pregestational maternal metabolic disorders affect progeny [29]. The inherited effects of pregestational maternal diet were especially pronounced in the pups of dams fed with LAWR in the context of a HFD. Since the observed effects in this study were due to a pregestational exposure of the dams to the various interventions, a worse scenario could play out in both parents and offspring that consume a particular rice cultivar as their staple food and also in parents who consume it during gestation.

Germination and high-amylose content appear to have additive mitigating effects on the impact of rice consumption on markers of obesity and IR. Statistically, germination played a greater role than amylose content in controlling the effects of rice consumption on weight gain and lipid profile as well as adiponectin levels in dams and pups. This may be related to the fact that the bioactive substances in germinated brown rice exert their effects through interaction with regulatory proteins. The physiological effect of high amylose content, on the other hand, is to reduce the magnitude of postprandial changes in glucose level and thus delay development of the downstream effects of chronic hyperglycaemia. This effect - unlike that of the bioactive constituents of germinated brown rice ‒ occurs over long time periods. The regression coefficient and interaction between the effects of high amylose content and germination (*p* < 0.05) - seen, for example in the effect of both doses of HAGBR on all parameters investigated - provides support for our hypothesis that promoting the consumption of germinated rice from high-amylose cultivars as an alternative to the current staple forms of rice would reduce the burden of type 2 diabetes by reducing predisposition to obesity and IR.

## Conclusion

We conclude from these results that for those who consume rice as a staple, choosing a high-amylose cultivar *and* consuming germinated rice would reduce predisposition to IR and obesity more than consuming rice with just one of these properties. Germination status had more impact on obesity and IR than amylose content. Consumption of rice as a staple increases predisposing to obesity and IR most when the form of rice chosen combines lack of fibre (white rice) and low amylose content.

## Supporting information

S1 Table Day 1 fasting blood glucose levels of 120 nulliparous rats. (DOCX)

S2 Table Day 3 fasting blood glucose levels of 120 nulliparous rats. (DOCX)

S3 Table Weight of pups resulting from dams fed with normal pellet related diet. (DOCX)

S4 Table Weight of pups resulting from dams fed with high fat related diet. (DOCX)

S1 Fig 2 h area under curve after oral glucose tolerance test on pups resulting from dams fed with NP related interventions. AC: acarbose; GBR: germinated brown rice; HAGBR: high amylose germinated brown rice; HAWR: high amylose white rice; LAGBR: low amylose germinated brown rice; LAWR: low amylose white rice; NP: normal standard pellet. No significant difference at p<0.05.
(DOCX)

S2 Fig 2 h area under curve after oral glucose tolerance test on pups resulting from dams fed with HFD related interventions. AC: acarbose; GBR: germinated brown rice; HAGBR: high amylose germinated brown rice; HAWR: high amylose white rice; HFD: high fat diet; LAGBR: low amylose germinated brown rice; LAWR: low amylose white rice. No significant difference at p<0.05.
(DOCX)
