# Supplementary material for: Predisposition to insulin resistance and obesity due to staple consumption of rice: Amylose content versus germination status
Source: PLoS One. 2017 Jul 20;12(7):e0181309. doi: 10.1371/journal.pone.0181309 (PMC5519073; doi:10.1371/journal.pone.0181309)
Supplement: S1 Table — (DOCX) [file pone.0181309.s001.docx]

S1 Table: Day 1 fasting blood glucose levels of 120 nulliparous rats.

|  |  |  |  | fasting blood glucose level (mmol/L) | | | | Day 1 |  |  |  |  |
| --- | --- | --- | --- | --- | --- | --- | --- | --- | --- | --- | --- | --- |
| rats 1-12 | 5 | 3.9 | 4.4 | 4.6 | 4.4 | 4.5 | 4.6 | 5 | 5.2 | 4 | 4.2 | 4.1 |
| rats 13-24 | 4 | 4.5 | 4.2 | 4.1 | 4.6 | 5.1 | 5 | 4.9 | 4.3 | 5.1 | 5 | 4.6 |
| rats 25-36 | 4.6 | 3.9 | 5.1 | 4.8 | 4 | 5.1 | 5 | 4.9 | 4.2 | 4.5 | 4.6 | 4.5 |
| rats 37-48 | 4.2 | 4.1 | 5.2 | 4 | 4.2 | 4.1 | 5.2 | 4 | 4.1 | 5.1 | 4.9 | 5.2 |
| rats 49-60 | 5.1 | 5.2 | 4 | 4.9 | 4.5 | 5 | 4.6 | 4.7 | 4.4 | 5 | 4.8 | 3.9 |
| rats 61-72 | 3.9 | 4.5 | 4.9 | 5.2 | 4.8 | 4 | 3.9 | 5 | 4.2 | 4.1 | 4.7 | 5.1 |
| rats 73-84 | 4.6 | 4 | 4.7 | 3.9 | 4.4 | 4 | 4.9 | 4.4 | 5.2 | 4.8 | 5 | 3.9 |
| rats 85-96 | 5 | 5.2 | 4.9 | 5.1 | 4.6 | 4.9 | 3.9 | 5.1 | 4.6 | 4.7 | 4.5 | 5.2 |
| rats 97-108 | 3.9 | 4.8 | 5 | 4.4 | 4.7 | 4.3 | 5.2 | 4.7 | 4.8 | 5.2 | 4.9 | 5.1 |
| rats 109-120 | 4 | 4.5 | 5.1 | 4.2 | 4.1 | 4.5 | 4.6 | 5 | 3.9 | 4.6 | 4.2 | 4.1 |
