# Supplementary material for: Predisposition to insulin resistance and obesity due to staple consumption of rice: Amylose content versus germination status
Source: PLoS One. 2017 Jul 20;12(7):e0181309. doi: 10.1371/journal.pone.0181309 (PMC5519073; doi:10.1371/journal.pone.0181309)
Supplement: S3 Table — (DOCX) [file pone.0181309.s003.docx]

S3 Table. Weight of pups resulting from dams fed on standard chow-based diet

| GROUPS (pups) | W0 | W1 | W2 | W3 | W4 | W5 |
| --- | --- | --- | --- | --- | --- | --- |
| SC | 5.5 | 15 | 30 | 50 | 84 | 108 |
| SC+50%HAGBR | 6 | 17 | 31 | 52 | 90 | 103 |
| SC+50%LAGBR | 5.8 | 16 | 32 | 53 | 87 | 107 |
| SC+50%HAWR | 5.3 | 14 | 30 | 53 | 85 | 104 |
| SC+50%LAWR | 5.2 | 14 | 29 | 50 | 88 | 104 |
| SC+50%LAWR+AC | 5 | 15 | 29 | 49 | 88 | 107 |
| SC+25%HAGBR | 5 | 14 | 30 | 51 | 89 | 107 |
| SC+25%LAGBR | 5.8 | 15 | 31 | 50 | 88 | 102 |
| SC+25%HAWR | 5.5 | 16 | 32 | 51 | 90 | 102 |
| SC+25%LAWR | 5.7 | 16 | 33 | 53 | 87 | 101 |
