# Supplementary material for: Predisposition to insulin resistance and obesity due to staple consumption of rice: Amylose content versus germination status
Source: PLoS One. 2017 Jul 20;12(7):e0181309. doi: 10.1371/journal.pone.0181309 (PMC5519073; doi:10.1371/journal.pone.0181309)
Supplement: S4 Table — (DOCX) [file pone.0181309.s004.docx]

S4 Table: Weight of pups resulting from dams fed on high-fat diet-based interventions

| GROUPS (pups) | W0 | W1 | W2 | W3 | W4 | W5 |
| --- | --- | --- | --- | --- | --- | --- |
| HFD | 5.3 | 14 | 29 | 53 | 87 | 106 |
| HFD+50%HAGBR | 5.4 | 15 | 29 | 52 | 89 | 108 |
| HFD+50%LAGBR | 5.6 | 17 | 31 | 49 | 95 | 105 |
| HFD+50%HAWR | 5.6 | 15 | 30 | 49 | 94 | 107 |
| HFD+50%LAWR | 5.7 | 15 | 30 | 50 | 93 | 103 |
| HFD+50%LAWR+AC | 5.9 | 15 | 29 | 52 | 89 | 105 |
| HFD+25%HAGBR | 5.4 | 16 | 32 | 51 | 93 | 110 |
| HFD+25%LAGBR | 5.9 | 15 | 33 | 50 | 88 | 105 |
| HFD+25%HAWR | 5.3 | 16 | 32 | 51 | 93 | 109 |
| HFD+25%LAWR | 5.5 | 14 | 33 | 49 | 91 | 105 |
