# Supplementary material for: Predisposition to insulin resistance and obesity due to staple consumption of rice: Amylose content versus germination status
Source: PLoS One. 2017 Jul 20;12(7):e0181309. doi: 10.1371/journal.pone.0181309 (PMC5519073; doi:10.1371/journal.pone.0181309)
Supplement: S2 Fig — AC: acarbose; GBR: germinated brown rice; HAGBR: high amylose germinated brown rice; HAWR: high amylose white rice; HFD: high fat diet; LAGBR: low amylose germinated brown rice; LAWR: low amylose white rice. No significant difference at p<0.05. (DOCX) [file pone.0181309.s006.docx]

2 h area under curve after oral glucose tolerance test on pups resulting from dams fed with HFD based interventions. AC: acarbose; GBR: germinated brown rice; HAGBR: high amylose germinated brown rice; HAWR: high amylose white rice; HFD: high-fat diet; LAGBR: low amylose germinated brown rice; LAWR: low amylose white rice. No significant difference at p<0.05.
